# Insights into the Versatility of Using Atomic Absorption Spectrometry in Antibacterial Research

**DOI:** 10.3390/molecules29133120

**Published:** 2024-06-30

**Authors:** David Krüger, James T. P. Matshwele, Muhammad Dauda Mukhtar, Daniel Baecker

**Affiliations:** 1 Department of Pharmaceutical Biology, Institute of Pharmacy, Freie Universität Berlin, Königin-Luise-Straße 2+4, 14195 Berlin, Germany; d.krueger@fu-berlin.de; 2 Department of Chemistry, Faculty of Science, University of Botswana, Private Bag 0704, Gaborone, Botswana; matshwelej@ub.ac.bw; 3 Department of Pharmaceutical Microbiology and Biotechnology, Faculty of Pharmaceutical Sciences, Bayero University Kano, Gwarzo Road, Kano PMB 3011, Nigeria; mdmukhtar.mcb@buk.edu.ng; 4 Department of Pharmaceutical and Medicinal Chemistry, Institute of Pharmacy, Freie Universität Berlin, Königin-Luise-Straße 2+4, 14195 Berlin, Germany

**Keywords:** antibiotics, atomic absorption spectrometry, cephalosporin, complexation, fluoroquinolone, mode of action, molecular absorption spectrometry, nanoparticles, quantification, resistance

## Abstract

The ongoing development of bacterial resistance to antibiotics is a global challenge. Research in that field is thus necessary. Analytical techniques are required for such a purpose. From this perspective, the focus was on atomic absorption spectrometry (AAS). Although it is old, AAS often offers unexpected potential. Of course, this should be exploited. The aim was therefore to demonstrate the versatility of the technique in antibacterial research. This is illustrated by various examples of its practical application. AAS can be used, for example, to confirm the identity of antibacterial compounds, for purity controls, or to quantify the antibiotics in pharmaceutical preparations. The latter allowed analysis without laborious sample preparation and without interference from other excipients. In addition, AAS can help elucidate the mode of action or resistance mechanisms. In this context, quantifying the accumulation of the antibiotic drug in the cell of (resistant) bacteria appears to play an important role. The general application of AAS is not limited to metal-containing drugs, but also enables the determination of some organic chemical antibiotics. Altogether, this perspective presents a range of applications for AAS in antibacterial research, intending to raise awareness of the method and may thus contribute to the fight against resistance.

## 1. Introduction

The issue of increasing resistance towards antibiotic drugs is a global challenge. The factors behind the increase in resistant bacteria are manifold and include, for example, the excessive or incorrect use of antibiotics [1]. Counterfeit medicines can also contribute to this problem [2]. The development of new antibiotics, taking into account the “one-health approach”, plays a pivotal role in curbing the progression of resistance [3]. Drug candidates with alternative lead structures may prevent cross-resistance. Novel drug classes addressing completely new targets, however, can rule out the occurrence of further resistance for the time being, but provide the desired therapeutic effect against bacteria.

Recently, metal-based antibiotics (metalloantibiotics) have emerged as a promising strategy for example [4]. Appropriate methods are required for the physicochemical, analytical, and pharmacological characterization as well as for the understanding of the antibacterial mechanisms of the new drug candidates [5].

One analytical technique in this context is atomic absorption spectrometry (AAS). Without justification, it is often described as old-fashioned and therefore uninteresting compared to using inductively coupled plasma mass spectrometry (ICP-MS). Recent research in pharmaceutical sciences shows the value of AAS. Examples include the analysis of medicinally interesting plants [6], the screening of drug candidates that activate the K_v_7.2/3 channel [7], or characterizing the biological profile of anticancer drugs [8], among others.

Advantages of AAS over ICP-MS were mentioned in a previous publication and include significantly lower acquisition and operating costs [9]. The refinement of AAS to molecular absorption spectrometry (MAS) extends the scope of the technique for metals to include some non-metals [10]. This enables the analysis of organic antibiotics that do not contain metals. The suitability of MAS for oligopeptides was also recently demonstrated [11]. This could, for instance, open the way for the analysis of peptide-based antibiotics, even if further research remains to be explored in this regard.

Based on these facts, the aim of this perspective is to present particular examples of how to use AAS in connection with antibiotic research. By means of the selected practical applications, the versatility of the technique will be demonstrated. This in turn can raise the awareness of scientists to utilize AAS in research in the general field of combating the rising resistance to antibiotic drugs.

## 2. Examples of Using AAS in Antibacterial Research

An analytical technique can be considered versatile if, on the one hand, it is feasible to detect various analytes, and, on the other hand, if it captures these analytes from different sample matrices. If the analytical procedure can be used for a number of diverse applications, this also extends its scope. What follows is the provision of examples of where AAS can be exploited purposefully in antibiotic research.

**Scheme 1:**
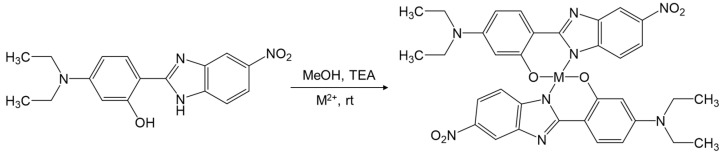
Complexation of the ligand 5-(diethylamino)-2-(5-nitro-1*H*-benzimidazol-2-yl)phenol in methanol (MeOH), supplemented with triethyl amine (TEA, 3–4 drops), with metal salts (M^2+^) at room temperature (rt) to form the respective Co(II), Cu(II), Fe(II), and Ni(II) complexes [12].

Chemical synthesis takes place at the beginning of antibacterial drug development. AAS can already be used at this stage to confirm the chemical structures of the newly synthesized compounds. This approach was followed in the work of Padalkar et al. [12]. Cobalt(II), copper(II), iron(II), and nickel(II) complexes were synthesized using cobalt(II) acetate, copper(II) acetate, iron(II) sulfate heptahydrate, and nickel(II) acetate, respectively. 5-(Diethylamino)-2-(5-nitro-1*H*-benzimidazol-2-yl)phenol served as the ligand (see Scheme 1). The resulting compounds were screened for their potential as antibacterial drugs. The biological activity of the complexes was investigated against strains of *Escherichia coli* and *Staphylococcus aureus*.

AAS was used to confirm the successful complexation between the metal cation and ligand in a stoichiometric ratio of 1:2. For this purpose, samples were weighed, dissolved, appropriately diluted, and analyzed using flame atomic absorption spectrometry (F AAS). The measurements revealed a proportion of Co(II), Cu(II), Fe(II), and Ni(II) in the complex of 1.94, 1.89, 1.87, and 1.93, respectively. This proved the formation of the complexes with the proposed stoichiometry (1:2). All the four complexes exhibited a minimum inhibitory concentration (MIC) equal to or even lower than the used reference streptomycin.

Apart from testing the identity of an antibiotic compound, AAS can also be used for purity control. In the quality studies published by de Paula et al., electrothermal atomic absorption spectrometry (ET AAS) was used to determine possible contamination of tablets containing the antibiotics ciprofloxacin (fluoroquinolone) or cephalexin (first-generation cephalosporin) with inorganic compounds (i.e., copper and manganese) [13]. Contamination with high levels of copper and manganese can cause neurotoxicity and organ impairment [14,15]. These can ultimately be present in the drugs due to the raw material of organic synthesis. The authors state that using AAS is simple and fast as well as advantageous owing to high selectivity (element-characteristic absorption wavelength) and sensitivity. The method is therefore recommended for routine analyses [13].

In addition to metal complexes, some inorganic materials (metal ions or metal oxides) themselves also exhibit antimicrobial properties, e.g., silver and copper [16,17]. Such inorganic materials are commonly used to generate nanoparticles. The latter are frequently loaded with antibiotics. Such hybrid nanosystems represent a promising approach as new agents against bacteria [18].

Golmohamadi et al. produced chitosan-based nanoparticles [19]. During the synthesis procedure, the nanoparticles were loaded with clarithromycin. For this purpose, an appropriately formulated solution of the macrolide antibiotic (in order to avoid aggregation of the drug) was added to a solution of chitosan and sodium tripolyphosphate. The proportion of loading of the nanoparticles could thus be checked using AAS, although no details of the specific procedure were provided in the study. The test of antibacterial activity against *Staphylococcus aureus* documented a better effect of the nanoparticles loaded with clarithromycin than when the antibiotic was applied by itself.

AAS was employed in the study by Matai et al. to investigate the antibacterial activity of nanocomposites consisting of silver and zinc oxide (Ag-ZnO) [20]. The Ag-ZnO nanocomposites were synthesized. Their analytical characterization was carried out using AAS by determining the concentration of silver and zinc. The quantification of the nanocomposites (2 µg mL^−1^) resulted in 0.621 and 0.626 µg mL^−1^ of silver and zinc, respectively. This confirmed approximately equivalent proportions of the two components in the nanocomposite. In addition, AAS was utilized in this research work to verify the antibacterial mode of action. It is known that silver ions [21] and zinc ions [22] exert an antibacterial effect. Therefore, the release of these ions from the Ag-ZnO nanocomposites kept in water was monitored after different incubation periods [20]. Quantification was performed with F AAS. The evaluation mainly revealed the tendency that the release of ions increased with longer incubation times. The antibacterial activity against *Staphylococcus aureus* and *Escherichia coli* (green fluorescent protein expressing and resistant), indicated as MIC and minimum killing concentration (MKC), also followed the trend of rising concentration of released ions. Hence, for example, an interaction of the cations with the cell membrane (negatively charged) could be deduced as one mechanism of action. The investigations using AAS thus contributed to the elucidation of the antibacterial mode of action.

Determining the release of an antibacterial species was also implemented in the study of Zietz et al. [23]. In particular, upon impregnating the surfaces of medical implants with copper, the release of the respective Cu(II) ions as a result of exposure to media such as double-distilled water, human serum, or Dulbecco’s Modified Eagle cell culture medium (DMEM, supplemented with 10% of fetal calf serum and 1% of gentamicin) was investigated. Implants with different surface treatments (i.e., polishing, blasting with hydroxyapatite, and corundum blasting) were applied for the tests, as their roughness also plays a role for the release behavior. Since Cu(II) ions are said to have antimicrobial properties and to be compatible with cells, the coating of the implant surfaces is intended to reduce the formation of bacterial biofilms and subsequent infections. The latter can, for example, lead to a certain rejection of the implant. After incubation in medium, the supernatant was further processed. The released Cu(II) ions were stabilized with nitric acid (1%) and appropriately diluted to be finally measured via graphite furnace atomic absorption spectrometry (GF AAS, synonymous to ET AAS). Untreated implants (without copper coating) had no copper in the supernatant. The highest release of copper was found after immersion in DMEM, followed by the samples treated with human serum. Only very little release was induced by treating the implants with bidistilled water.

Burygin et al. investigated the influence of gold nanoparticles on the activity of gentamicin [24]. The antibacterial activity of this aminoglycoside antibiotic was tested against *Escherichia coli* K12 either in its pure form or additionally mixed with gold nanoparticles. Surprisingly, no significant difference was found. In order to further elucidate this phenomenon, the content of gold in the agar of the diffusion zones was determined. For this purpose, a defined piece was cut from the agar, ashed (sulphuric acid, 600–630 °C), dissolved in a mixture of hydrochloric and nitric acid, and quantified using AAS. The results showed the presence of gold in the agar/medium when free gold nanoparticles were applied. However, no gold could be identified when gold nanoparticles were mixed with gentamicin. Since the nanoparticles did not diffuse into agar/medium as measured using AAS, the lack of enhancement of the effect could be explained. As the activity was approximately the same, it was assumed that only a small proportion of gentamicin binds to the nanoparticles.

**Figure 1:**
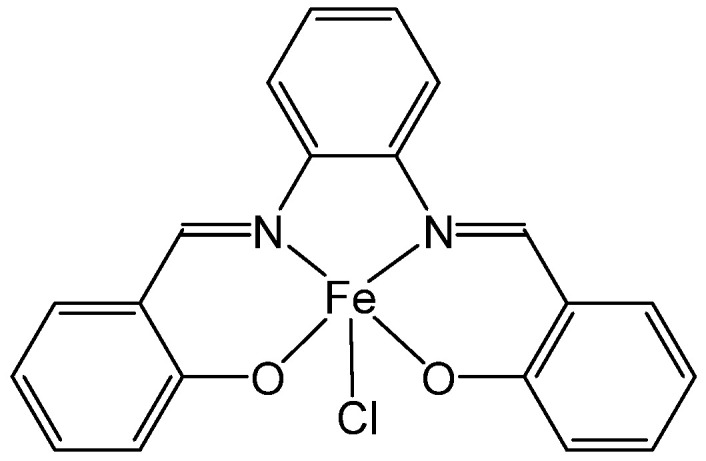
Chemical structure of chlorido[*N,N’*-bis(salicylidene)-1,2-phenylenediamine]iron(III) whose superior activity in Gram-positive bacteria compared to Gram-negative bacteria and whose greater effect in phosphate-buffered saline compared to Mueller Hinton Broth was elucidated with the aid of graphite furnace atomic absorption spectrometry.

The extent of binding in the manner of protein binding, as reported by Baecker et al. [25], also clarified the variable behavior of an antibiotic compound in different media. In this research work, a series of salen- and salophene-based compounds were evaluated for their activity against the Gram-positive bacteria *Staphylococcus aureus* and methicillin-resistant *Staphylococcus aureus* (MRSA) as well as the Gram-negative bacteria *Escherichia coli* and *Pseudomonas aeruginosa*. In particular, the compound chlorido[*N,N’*-bis(salicylidene)-1,2-phenylenediamine]iron(III) (see Figure 1) has emerged as a promising lead. This very active complex was investigated in kinetic time-kill tests with Mueller Hinton Broth (MHB) and phosphate-buffered saline (PBS). In PBS, the time to kill the bacteria was 10- to 14-times faster (testing three different concentrations) than in MHB. The possibility of decomposition of the compound by the proteins contained in MHB has previously been ruled out by the authors [26]. However, high protein binding was observed in MHB, limiting the amount of free, available drug [25]. GF AAS was used for the quantification. After incubation of the complex with MHB, the proteins were precipitated and the amount of free compound from the supernatant (indirect determination) was measured in terms of iron.

Interestingly, they found in their study that the compounds were inactive against the Gram-negative bacteria [25]. The induction of ferroptosis (lipid oxidation at the cytoplasmic membrane) was deduced as the mode of action against the Gram-positive bacteria, suggesting that the additional outer membrane of the Gram-negative bacteria could serve as a kind of barrier. Therefore, the accumulation of chlorido[*N,N’*-bis(salicylidene)-1,2-phenylenediamine]iron(III) in *Staphylococcus aureus* and *Escherichia coli* was quantified using GF AAS. The cellular uptake studies showed an approximately 5-fold higher uptake in the Gram-positive bacteria compared to the Gram-negative bacteria. The limited accessibility of the drug to the target, i.e., the cytoplasmic membrane, as determined using GF AAS explains its different potency.

AAS also made a contribution to elucidating the mechanism of action in the study by Syarifuddin et al. [27]. The ethyl acetate extract TE 325 of *Actinomycetes* bacteria (obtained from the rhizosphere of *Saccharum officinarum*) was examined with regard to its activity against *Escherichia coli*. Destruction of cell membrane integrity is considered to be the mechanism of action. The leakage of metal ions, which are relevant for the integrity of the membrane, can be assessed using AAS. Potassium ions (K^+^) in the cytoplasm contribute to the stability of the cell membrane permeability, while calcium ions (Ca^2+^) interact with the lipopolysaccharide layer and stabilize this structure. Therefore, K^+^ and Ca^2+^ were determined in the bacterial assay after incubation with the ethyl acetate extract TE 325. A rise in the amount of K^+^ outside the cell (if not precipitated by alkaloids contained in high extract concentrations) therefore indicated a disturbance of permeability and increased Ca^2+^ values suggested damage to the cell wall.

As an alternative to K^+^, rubidium ions (Rb^+^) can also be measured as a surrogate if the bacteria are provided with a Rb^+^-enriched growth medium [28]. If the integrity of the cell membrane is affected due to disruption, Rb^+^ ions leak out through the resulting pores or channels (ionophoric effect). This can be monitored using AAS and points to the mechanism of action. Antibiotics with an ionophore effect, i.e., the killing of bacteria through the formation of pores, include gramacidins [29]. AAS is thus particularly suitable for confirming the mode of action of derivatives of such antibiotics.

**Figure 2:**
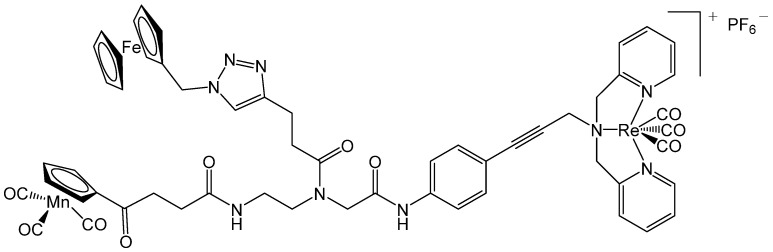
Chemical structure of the hetero-tri-organometallic complex FcPNA.

The extent of accumulation in different cellular compartments (i.e., cytosol, membrane, and cell wall) of *Bacillus subtilis* after incubation with the compound FcPNA was investigated by Wenzel et al. using GF AAS [30]. The measurements were based on the quantification of manganese. The studied drug FcPNA consists of a peptide–nucleic acid backbone connected with an alkyne linker bearing a (dipicolyl)Re(CO)_3_ core, and is substituted by a cymantrene (manganese cyclopentadienyl tricarbonyl) and a ferrocene (see Figure 2). After incubation of the bacterial cells with the complex, samples were washed several times with TRIS-EDTA (tris(hydroxymethyl)aminomethane and ethylenediaminetetraacetic acid) to eliminate freely attached manganese. The cells were then subjected to differential centrifugation, which resulted in fractions of the cytosol, membrane, and cell wall. These fractions were analyzed using GF AAS. The measurement revealed an approximately 10-fold higher enrichment in the membrane compared to the untreated reference. In addition, the manganese content in the membrane was about 6-times greater than in the cytosol and the cell wall. Since most of the compound FcPNA is found in the membrane and less in the other compartments, it can be concluded that the antibiotic effect of the drug is less due to a target in the cytosol or in the cell wall, but rather to an interaction with the membrane.

**Figure 3:**
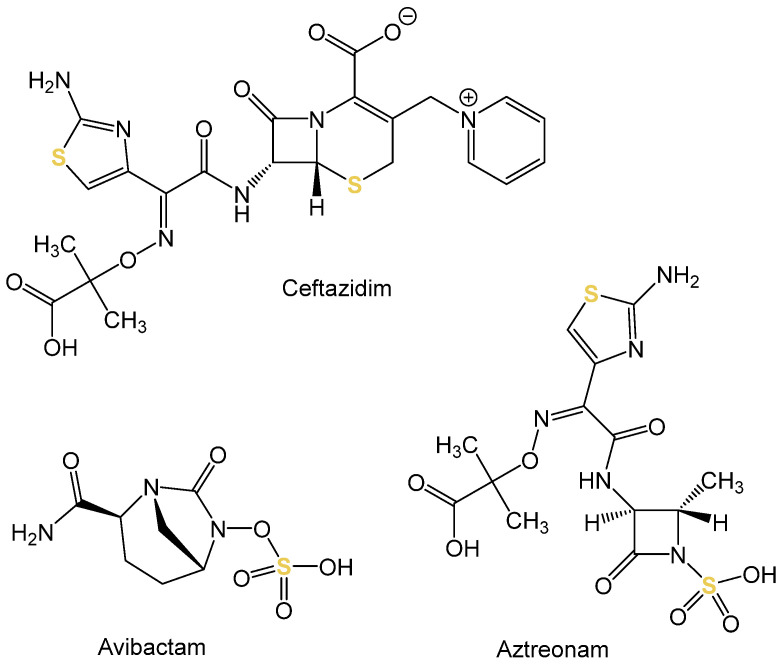
Chemical structures of the sulfur-containing drugs ceftazidime (third-generation cephalosporine), avibactam (non-β-lactam β-lactamase inhibitor), and aztreonam (monobactam antibiotic). The quantification was based on the sulfur atoms, which are highlighted in yellow.

In contrast to the contribution of AAS-based analysis to the elucidation of the mode of action, the spectroscopic technique can also provide support in shedding light on resistance mechanisms. Eger et al. investigated an extensively drug-resistant *Klebsiella pneumoniae* (sequence type 307) strain that exhibited resistance to the combination of the third-generation cephalosporin, i.e., ceftazidime, and the β-lactamase inhibitor avibactam [31]. There was also resistance to the monobactam aztreonam. In certain mutations of the ompK36 gene, the non-selective porin OmpK34 leads either to a narrowing in the diameter of the porin pore or even to its complete destruction. Consequently, this has an influence on the penetration of the antibiotics. Therefore, the uptake of the three sulfur-containing drugs (see Figure 3) into mutated strains (smaller pore diameter and loss of porin) was quantified. For the measurement, high-resolution continuous-source molecular absorption spectrometry (HR CS MAS) was employed using the graphite furnace sub-technique. The increase in cellular sulfur content as a result of treatment with the sulfurous antibiotics was based on the measurement of the absorption of the diatomic molecule carbon monosulfide (CS). The sulfur content of untreated bacteria served as a baseline. For the mutation with smaller pore diameter and destruction of the porin, respectively, uptake of ceftazidime was reduced by 88.4% and 71.3%, and for aztreonam by 32.6% and 37.9%, respectively. The uptake of avibactam was completely abolished (smaller diameter: reduction by 97.6% and complete depletion of porin: reduction by 99.3%). MAS thus provided valuable evidence to clarify the resistance mechanism.

In addition to mutations in certain genes that lead to the development of resistance, particular antibiotic resistance genes (ARGs) have also been described. ARGs are pervasive in agricultural soils due to the widespread farming with livestock as well as the application of sewage sludge and organic waste [32,33]. The study by Qi et al. focused on investigating the interactions between eight heavy metals (i.e., arsenic, cadmium, chromium, copper, lead, mercury, nickel, and zinc) and ARGs in soil samples [34]. For this purpose, the concentrations of the metals were determined using AAS. The results showed a significant correlation between some of the investigated heavy metals and ARGs. A cluster analysis of the data made it possible to draw conclusions about correlations. On the one hand, zinc, cadmium, and nickel were grouped together and, on the other, chromium, mercury, and arsenic with lead and copper. Even if trends can be derived, the coherences are diverse and other parameters (e.g., type of bacteria or antibiotics) also play a role. Specific soil remediation in line with the holistic “one-health approach” could help to reduce further evolution of resistance.

In the context of ARGs, it is known that metals can cause a selection pressure, which has a longer-lasting effect, as metals are not susceptible to short-lived decomposition compared to antibiotics [35]. Consequently, the co-presence of metals and antibiotics (for example as a result of sewage sludge manuring) can stimulate the development of resistance in populations of soil bacteria. The publication by Cheng et al. deals with a similar topic, in which the concentration of heavy metals and antibiotics in Chinese sewage sludges was determined [36]. After digestion with aqua regia (nitric acid (65%) and hydrochloric acid (37%), 1:3), the heavy metals in the samples were quantified using GF AAS. The most frequently occurring metal in the samples was zinc, followed by copper. Significantly lower concentrations were found for cadmium, chromium, lead, and nickel. In addition, 16 antibiotics were detected after extraction and analysis using liquid chromatography–tandem mass spectrometry (LC-MS/MS, electrospray ionization). Fluoroquinolones and tetracyclines were the most abundant, followed by sulfonamides. A significant correlation was found between the metals zinc and lead and antibiotics of the tetracycline class (with the exception of chlortetracycline). However, since the presence of heavy metals is not the main factor for the development of resistance, but the high concentration of antibiotics must also be viewed critically, it is concluded that further studies are needed to clarify these correlations in more detail. Then, the effects of manuring agricultural soil with sludge can also be better deduced.

**Figure 4:**
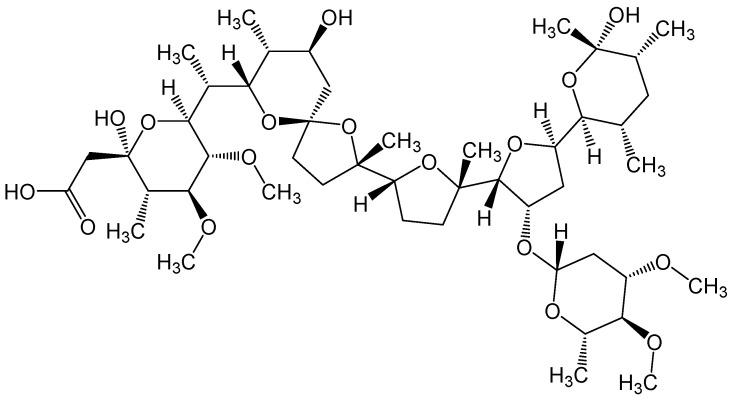
Chemical structure of maduramicin.

Another study with agricultural relevance is that of Johnson, in which the antibiotic maduramicin was detected and quantified in medicinal feeding stuff using AAS [37]. Maduramicin is a natural compound produced by *Actinomyces* and is applied in veterinary medicine in poultry, especially chicken and turkey, to prevent coccidiosis [38]. The trace analytical quantification of maduramicin is very challenging [37,39]. Due to many functional moieties and high molecular mass (M = 917.14 g mol^−1^, see Figure 4), gas chromatography (GC) is hardly feasible. The lack of a chromophore absorbing at λ ≥ 220 nm also makes conventional liquid chromatography (LC) with UV-detection impossible. However, AAS was coupled to LC so that maduramicin was measured with high sensitivity and specificity after complexation with sodium ions.

Coordination with metals, which can be quantified using AAS, offers a broad range of possibilities for the (indirect) detection of organic chemical antibiotics that genuinely do not contain metals. In order for such antibiotics to serve as ligands for complexation, they must have functional groups with free electrons (i.e., electron pair donor (Lewis acid), such as alcohols or carbonyls). This is the case with tetracyclines, for example. They exert their antibiotic effect by inhibiting the protein biosynthesis of the bacteria at the ribosomes. The complexing property can reduce the effect if, for example, dairy products (due to the high content of Ca^2+^) are not taken at a sufficient time interval from the medication.

**Figure 5:**
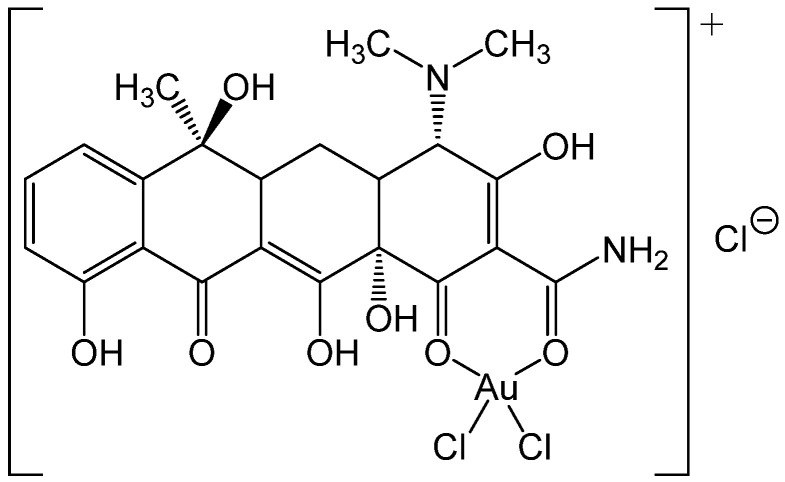
Proposed chemical structure of the complex due to the chelation of tetracycline with Au(III).

However, the affinity for complex formation can be utilized for analytical purposes. This was described in the publication by Abdulghani et al. [40]. The antibiotic tetracycline was quantified in capsules using F AAS as a result of complexation with Au(III). The complex formation of tetracycline hydrochloride and Au(III) followed a molar ratio of 1:1 (see Figure 5), which was confirmed with further analytical methods. This complex was finally analyzed using F AAS which allowed for the quantification of the antibiotic in capsules. The analytical method had a limit of detection (LOD) of 0.0997 µg mL^−1^ and showed linearity in the range 5–30 µg mL^−1^. The correlation coefficient was 0.9967 and the recovery amounted to 104.85%. The developed method was compared with a UV-Vis spectrophotometric method and with high-performance liquid chromatography (HPLC). The recoveries were similar, but the LOD was significantly lower for the F AAS-based procedure (UV-Vis: 0.7403 µg mL^−1^, HPLC 2.647 µg mL^−1^). This makes the AAS measurements distinctly more sensitive and a competitive method.

**Scheme 2:**
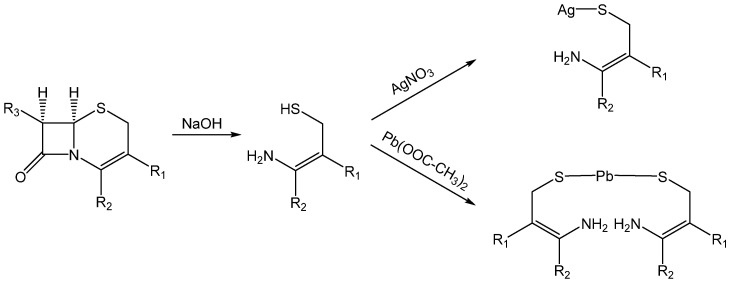
Chemical approach for the evaluation of cephalosporins after NaOH-based hydrolysis and subsequent complexation.

In the study by Ayad et al., the two antibiotics cefuroxime and cefotaxime (cephalosporins of the second and third generation, respectively) were quantified using AAS in both a direct and indirect manner as a result of coordination with silver(I) or lead(II) [41]. However, it was not the drugs themselves that served as ligands for complexation, but the products formed during alkali-hydrolysis (see Scheme 2). The ligand (thiol group) was unveiled from the derived cepham core (i.e., 5-thia-1-azabicyclo[4.2.0]oct-4-en-8-one). After hydrolysis with sodium hydroxide (NaOH), neutral aqueous solutions of the products were reacted with silver nitrate and lead acetate. The resulting precipitates were measured regarding their content of silver(I) or lead(II) using AAS (direct method). In addition, the unreacted amount of metal ions in the filtrate was also analyzed using AAS (indirect method).

In detail, the precipitate was diluted in ammonia (NH_3_) after reaction with silver nitrate. In the case of coordinating with lead acetate, the precipitate was dissolved in hydrochloric acid (HCl) for AAS-based analysis. In both cases, the filtrate was adjusted with bidistilled water. The developed methods showed good performance in the analysis of pharmaceutical preparations (i.e., vials containing either of the two cephalosporins). The recoveries for both methods were in the range from 98.73% to 101.36% (see Table 1), thus documenting high accuracy. In addition, the low standard deviations document good precision.

The publication by Dhahir et al. reports that the cephalosporin cefixime (third generation) was quantified in capsules using F AAS, but without hydrolysis [42]. For this purpose, a palladium(II)–cefixime precipitate (molar ratio 1:1) was generated with palladium chloride. In addition to its simplicity, sensitivity, accuracy, and precision, the method is praised as a green chemistry method. Compared to HPLC, no large quantities of organic solvents are required.

The cephalosporin antibiotics cephalexin and cephradine, both belonging to the first generation, were quantified in the study by Al-Ghannam using AAS as a result of complexation with Reinecke’s salt (ammonium tetrathiocyanatodiamminochromate(III) monohydrate, NH_4_[Cr(NCS)_4_(NH_3_)_2_]∙H_2_O) [43]. For this purpose, aqueous solutions of the cephalosporins were reacted with Reinecke’s salt in an acidic medium to yield purple-colored ion-pair complexes. The analysis was based on chromium. On the one hand, it was performed in a direct manner of the precipitated complex and on the other indirectly by residual of Reinecke’s salt in the filtrate. The method was used to quantify the two pure drugs and also in pharmaceutical preparations (cephalexin: capsules, tablets; cephradine: capsules, vials). The recovery rates when quantifying the free drugs cephalexin monohydrate and cephradine were almost exactly 100% (see Table 2), independent of either direct or indirect determination. The same is true for the analysis of these two cephalosporins in dosage forms. The latter results show that there appears to be no significant interference with other ingredients of the pharmaceutical preparations. The method is therefore recommended for routine analysis.

Precipitation of organic chemical antibiotics with Reinecke’s salt was also used in the study by Ragab et al., followed by chromium-based quantification through F AAS [44]. In particular, the fluoroquinolones ciprofloxacin, enrofloxacin, norfloxacin, and ofloxacin were the subjects of the investigations. These drugs formed steady precipitates that are poorly soluble in water but were dissolved in acetone for the spectrometric measurements. The elaborated method had a comparably good quality compared to the official analysis (i.e., titration with perchloric acid). Thus, it was applied to the analysis of the fluoroquinolones in pharmaceutical preparations (i.e., tablets, eye drops, and injection). Valuable analytical parameters were achieved, such as recoveries ranging from 99.0% to 101.0%.

The publication by Salem also described the assessment of fluoroquinolones according to the generation of complexes as a result of reaction with Reinecke’s salt in an acidic environment [45]. Chromium-based quantification using F AAS was also performed either directly (precipitate) or indirectly (filtrate). A total of ten different fluoroquinolones (and pharmaceutical dosage forms of some of them), i.e., amifloxacin, ciprofloxacin HCl (tablets and drops), difloxacin HCl, enoxacin, enrofloxacin, levofloxacin (tablets), lomefloxacin HCl, norfloxacin (tablets), ofloxacin (tablets), and pefloxacin MsO (tablets and ampoules) were included in the study. The free drugs achieved recovery rates of 98.98–100.30% (direct method) and 98.96–100.50% (indirect method), thus documenting the competitiveness of the AAS-based method (see Table 3). This is also evident for the recovery when the compounds were present in pharmaceutical formulations (direct method: 98.97–100.99%; indirect method: 98.88–100.99%). Good precision is ensured with low standard deviations (≤0.70%). There are no differences in the quality of the two approaches, i.e., direct versus indirect determination.

As a follow-up, in a further publication by Salem, the ten fluoroquinolones mentioned above were quantified using AAS as a result of precipitation in a slightly alkaline alcoholic solution (pH = 8.1) with the aid of cobalt sulphate [46]. The measurements were based on the absorption of cobalt. Here, too, a direct approach (dilution of the precipitate in acetic acid and water) and an indirect manner (dilution of the filtrate with water) were followed. The method for quantification of the pure drugs again showed recovery rates close to 100% (direct: 98.98–100.00%; indirect: 98.05–99.99%). Such a good performance of the analytical procedure was also found for the determination of the antibiotics in pharmaceutical preparations (direct: 98.00–102.00%; indirect: 96.87–101.98%). This indicates that there is no adverse interference with excipients with respect to quantification. In addition to this good accuracy, low standard deviations (≤0.94%) again document high precision. In addition, prepared samples of human urine and plasma were spiked with the antibiotics in this study. In these cases, too, both the direct and indirect methods were suitable for reliable quantification. Spiked urine samples had recovery rates of 98.92–101.75% and 98.29–101.98% for the direct and indirect approach, respectively, and for the spiked plasma samples, 98.87–102.01% and 98.28–102.00%.

**Figure 6:**
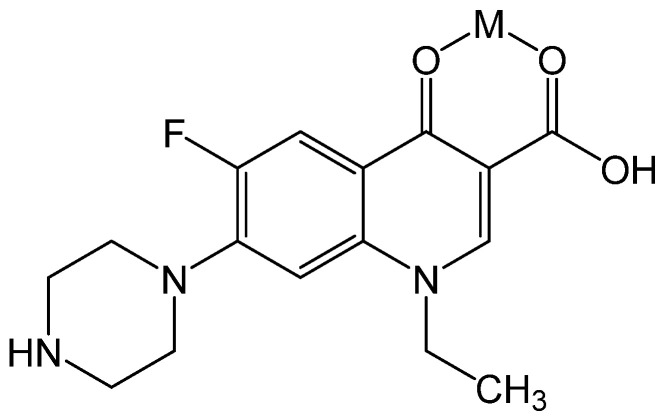
General chemical structure of the complex due to coordination of norfloxacin with metal ions (M, i.e., Cu(II) and Fe(III)).

Another way of quantifying the fluoroquinolone norfloxacin via complexation and measurement with F AAS was initially proposed by Zhang et al. [47]. This is based on the formation of a complex with copper(II) or iron(III) in a stoichiometric ratio of 1:1 (see Figure 6). The method was used to quantify norfloxacin in capsules from different suppliers. Comparing the results of the AAS analysis with the procedure described in the pharmacopoeia (i.e., titrimetry with perchloric acid) evidenced that both methods are in good agreement. AAS determination has advantages over the official pharmacopoeia method. AAS is more sensitive, more precise, requires a smaller sample volume, and enables a higher sample throughput due to its rapidity. The authors thus suggested a routine use for pharmaceutical products.

**Figure 7:**
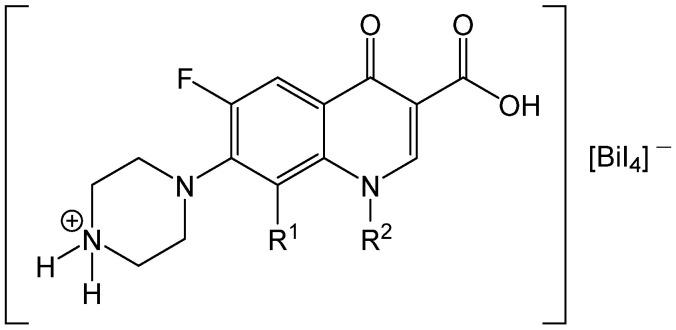
Proposed chemical structure of the ion-pair complex formed with tetraiodo bismuthate(III) and the fluoroquinolones ciprofloxacin (R^1^ = H, R^2^ = cyclopropyl), levofloxacin (R^1^ = O-CH(CH_3_)-CH_2_- = R^2^, *S*-configuration), and norfloxacin (R^1^ = H, R^2^ = ethyl) in acidic medium.

In the study by El-Brashy et al., the fluoroquinolones ciprofloxacin, levofloxacin, and norfloxacin were quantified via formation of a non-soluble ion-pair complex with excessive tetraiodo bismuthate(III) ([BiI_4_]^−^) in acidic medium and subsequent measurements with F AAS [48]. The acidic environment is necessary and leads to the protonation of the secondary amine (NHR_2_) of the piperazinyl residue. As a result, the antibiotic is present in cationic form and builds, with the anion [BiI_4_]^−^, an orange–red precipitate [NH_2_R_2_]^+^[BiI_4_]^−^ (see Figure 7) similar to Dragendorff’s reagent [49]. The separated precipitates were dissolved in acetone and measured for the bismuth content, as were the filtrates for the residual content of the bismuth-containing reagent [48].

This method was applied to quantify either the free antibiotics, pulverized tablets thereof, and urine samples of healthy volunteers spiked with the drugs. The recovery rates for the analysis of the pure drugs were close to 100% and thus document good accuracy (see Table 4). In addition, reliable precision is guaranteed due to low standard deviations (≤1.30%). The results were in good agreement with the reference method (titrimetric analysis). For the analysis of these fluoroquinolones in tablets, and as a result of spiking human urine, recoveries of almost 100% were also achieved. The latter, in particular, shows that interference with biological matrices can be ruled out with this type of quantification. This makes the AAS-based method a suitable choice.

## 3. Conclusions

The increasing resistance to antibiotics requires research in this field to understand the underlying mechanisms and to develop new drugs. This in turn demands analytical methods for the investigations. In this perspective, the technique of AAS was focused on, and the potentially unexpected versatility of its areas of application was demonstrated, pointing out a variety of practical examples.

On the one hand, AAS can contribute to the confirmation of the identity of metal-containing drugs. In addition, inorganic impurities originating from the raw materials of synthesis, for example, can be detected in organic chemical antibiotics, too.

Metalloantibiotics can be quantified based on their metal component. The determination of the content can also be applied to antibiotics with solely organic origin if they are detected after complexation with a metal. Such a procedure has already been successfully implemented many times for tetracyclines, cephalosporins, and fluoroquinolones. This approach is of particular interest when the antibiotic to be determined cannot be detected conventionally, for example due to a lack of chromophore (no UV-Vis detection with HPLC) or because of other physicochemical properties (high molecular mass or boiling point precludes GC analysis). For an AAS-based method as a result of chelation, however, complexing functional groups in the molecule would be necessary. If this is not available, quantification may be possible using MAS based on some non-metals. However, there is still a lot of potential for research in this particular area.

Several studies showed the great performance of AAS in the analysis of antibiotic drugs either in pure form, the quantification in pharmaceutical preparations, or even after spiking of human liquids (e.g., urine and plasma) with the drugs. The latter two analyses were carried out without special sample preparation and yielded very reliable recoveries. Thus, interferences with a matrix (i.e., other excipients in the dosage forms or biological liquids) can be excluded during the analysis. Because sample preparation, such as extraction, is not absolutely necessary, an AAS-based procedure seems more convenient than HPLC, for example. The omission of high quantities of organic solvents contributes to a certain aspect of green chemistry of AAS.

In addition, quantification using AAS can support the characterization of the pharmacological profile of antibiotic species. For example, the release of antibacterial metal ions from nanoparticles can be monitored as a part of the effect. An excessive release of physiological cations from the bacterial cell as a result of drug treatment indicates a mechanism of action with an influence on membrane integrity. AAS has also been used in studies investigating the binding of metallodrugs to protein. The quantification of the uptake of such compounds in bacterial cells or the differentiation into localization in various cellular compartments can complement the pharmacological profiling.

Since limited uptake or increased efflux of antibiotics from the cell represents an essential resistance mechanism, the quantification of accumulation also plays an interesting role in this context. In connection with resistance, correlations between the occurrence of ARGs and the content of certain metal ions have been documented, for example in soil. Again, the determination was based on AAS.

The areas of application of AAS are more diverse than one might initially think. This is apparent from the examples provided in this perspective, even if the authors often provide no further details on how to perform the respective analysis. However, AAS is a selective (characteristic wavelengths of analytes), accurate (reliable recoveries), and highly sensitive (very low LOD) method. Apart from this analytical performance, measurements are quite fast and often without elaborate sample preparation. This enables a high sample throughput and makes AAS suitable for quality controls. Compared to the competing alternative technique ICP-MS, AAS is less expensive. This is another reason to make it appropriate for routine analysis and especially suitable for quality control laboratories in developing countries. Since the application of AAS is not limited to pure metal-containing compounds, but also organic chemical antibiotics can be assessed (indirectly), this analytical technique has great potential. It is to be hoped that this capacity will be exploited in antibacterial research wherever possible.

## Figures and Tables

**Table 1:** Recoveries (±standard deviation) of the quantification of cefuroxime and cefotaxime via silver(I)- and lead(II)-based AAS after alkaline hydrolysis as reported by Ayad et al. [41].

Metal Ion	Silver (I)		Lead (II)	
Method	Direct	Indirect	Direct	Indirect
Cefotaxime	100.52 ± 1.04%	98.91 ± 1.03%	99.89 ± 0.91%	98.73 ± 0.92%
Cefuroxime	100.67 ± 1.11%	101.01 ± 1.15%	100.28 ± 0.69%	101.36 ± 0.73%

**Table 2:** Recoveries (±standard deviation) of the quantification of cephalexin and cephradine via chromium(III)-based AAS as reported by Al-Ghannam [43].

Drug	Direct Method	Indirect Method
Cephalexin monohydrate	99.39 ± 0.54%	100.04 ± 0.35%
Cephradine	99.77 ± 0.61%	99.96 ± 0.52%
Cephalexin capsules	99.25 ± 0.69%	99.55 ± 0.84
Cephalexin tablets	100.10 ± 0.59%	99.94 ± 0.66%
Cephradine capsules	99.45 ± 0.87%	100.15 ± 0.97%
Cephradine vials	99.74 ± 0.75%	99.90 ± 0.82%

**Table 3:** Recoveries (±standard deviation) of the quantification of fluoroquinolones via chromium(III)-based AAS as reported by Salem [45].

Drug	Direct Method	Indirect Method
Amifloxacin	98.98 ± 0.30%	99.35 ± 0.54%
Ciprofloxacin HCl	99.21 ± 0.60%	99.00 ± 0.60%
Difloxacin HCl	100.30 ± 0.45%	99.95 ± 0.42%
Enoxacin	99.04 ± 0.62%	99.01 ± 0.63%
Enrofloxacin	100.09 ± 0.63%	99.98 ± 0.59%
Levofloxacin	99.93 ± 0.53%	100.21 ± 0.52%
Lomefloxacin HCl	99.85 ± 0.54%	100.05 ± 0.49%
Norfloxacin	100.00 ± 0.53%	99.98 ± 0.55%
Ofloxacin	99.09 ± 0.51%	98.96 ± 0.59%
Pefloxacin MsO	99.98 ± 0.49%	100.50 ± 0.48%
Ciprofloxacin tablets	99.00 ± 0.62%	99.99 ± 0.61%
Ciprofloxacin tablets	99.59 ± 0.40%	99.99 ± 0.51%
Ciprofloxacin drops	99.89 ± 0.50%	100.04 ± 0.49%
Levofloxacin tablets	98.97 ± 0.55%	99.54 ± 0.51%
Norfloxacin tablets	100.05 ± 0.63%	100.00 ± 0.63%
Norfloxacin tablets	100.29 ± 0.55%	100.53 ± 0.49%
Ofloxacin tablets	100.05 ± 0.66%	99.98 ± 0.70%
Ofloxacin tablets	100.99 ± 0.44%	100.99 ± 0.43%
Pefloxacin tablets	99.00 ± 0.45%	98.88 ± 0.40%
Pefloxacin ampoules	100.01 ± 0.46%	100.11 ± 0.50%

**Table 4:** Recoveries (±standard deviation) of the quantification of fluoroquinolones via bismuth(III)-based AAS as reported by El-Brashy et al. [48].

Drug	Direct Method	Indirect Method
Ciprofloxacin	100.05 ± 0.47%	100.38 ± 1.30%
Levofloxacin	99.98 ± 0.33%	100.06 ± 1.26%
Norfloxacin	100.02 ± 0.49%	100.18 ± 1.18%

## Data Availability

Data sharing is not applicable.
